# The osteogenesis of bacterial cellulose scaffold loaded with fisetin

**DOI:** 10.22038/IJBMS.2018.25465.6296

**Published:** 2018-09

**Authors:** Elahe Vadaye Kheiry, Kazem Parivar, Javad Baharara, Bibi Sedigheh Fazly Bazzaz, Alireza Iranbakhsh

**Affiliations:** 1 Department of Biology, Science and Research Branch, Islamic Azad University, Tehran, Iran; 2 Research Center for Animal Development and Applied Biology, Mashhad Branch, Islamic Azad University, Mashhad, Iran; 3 Biotechnology Research Center, Institute of Pharmaceutical Technology, Mashhad University of Medical Sciences, Mashhad, Iran; 4 School of Pharmacy, Mashhad University of Medical Sciences, Mashhad, Iran

**Keywords:** Bacterial cellulose, Fisetin, Mesenchymal stem cells OCN, OPN

## Abstract

**Objective(s):**

Bacterial cellulose (BC) has applications in medical science, it is easily synthesized, economic and purer compared to plant cellulose. The present study aimed to evaluate BC, a biocompatible natural polymer, as a scaffold for the bone marrow mesenchymal stem cells (BMSCs) loaded with fisetin, a phytoestrogen.

**Materials and Methods:**

BC hydrogel scaffold was prepared from *Gluconaceter xylinus* and characterized through scanning electron microscopy (SEM). Biocompatibility of BC was measured by MTT assay, BMSCs were obtained from femur of rat and the osteogenic potential of the BC scaffold cultured with BMSCs and loaded with fisetin, was investigated by measuring the alkaline phosphatase (ALP) activity, alizarin red staining (ARS) and real-time PCR in terms of osteoblast-specific marker, osteocalcin (OCN) and osteopontin (OPN).

**Results:**

Biocompatibility results did not show any toxic effects of BC scaffold on BMSCs, while it increased cell viability. The data showed that BC loaded fisetin differentiated BMSCs into osteoblasts as demonstrated by ALP activity assays and ARS *in vitro*. Moreover, results from gene expression assay showed the expression of OCN and OPN genes was increased in cells that were seeded on the BC scaffold loaded with fisetin.

**Conclusion:**

According to the results of the present study, BC loaded with fisetin is an effective strategy to promote osteogenic differentiation and a proper localized delivery system, which could be a potential candidate in bone tissue engineering.

## Introduction

Bones are part of the skeletal system that is generated through the process of bone formation; osteogenesis begins during the prenatal development and continues throughout adulthood. A wide array of factors are involved in osteogenesis, such as hormones, growth factors, signaling pathways, and transcription factors (1). Bone is a dynamic tissue that constantly changes and has the capacity for restoration and regeneration (2). Growth of new cells and replacement of old cells enhances bone health and repairs small injuries and mechanical damages. However, treatment of bone diseases and abnormalities such as trauma and the abnormal birth defects is quite challenging. Remedy of these diseases is possible through the induction of osteoblast function. Furthermore, allograft, autograft, and use of artificial structures, have proven effective in the treatment of these diseases (2, 3). Engineered bone tissue using scaffold has been viewed as a possible substitute for the conventional use of bone grafts, due to unlimited supply and no transmission of disease (4). The aim of bone tissue engineering is induction of regeneration of new functional bone through the synergistic combination of biomaterials, cells, and factor therapy (4). Bacterial cellulose (BC) is one of the main biological materials used as a scaffold in bone tissue engineering (5). BC is derived from bacteria and grows in liquid sugar matrix, as a product of the extracellular bacterial cell wall without the structural components, with its specific features. BC has more applications in regenerative medicine compared to plant cellulose, some of these features include high purity, high tensile strength, potent and elastic modulus, easy purification, biocompatibility, and biofunctionality (6). Moreover, BC has been frequently used in previous *in vitro* and *in vivo* studies in vascular stents, wound dressing and differentiation of chondrocytes and osteocytes (7). BC fibers have a similar structure to bone collagens (3). Also in bone engineering, the used cells are the main factor. BMSCs are a population of self-renewing pluripotent cells with cellular treatment ability in tissue production (8). To increase the differentiation of stem cells into bone cells, growth factors including platelet-derived growth factor (PDGF), bone morphogenetic proteins (BMPs), and transforming growth factor-β (TGF-β) showed promising effects (9). However, the high cost and rapid degradation of such expensive growth factors limited their use (10). Therefore, in recent years the need to expand alternative lower cost, high capacity osteogenic inducers other than growth factors through the use of natural products especially medicinal plants increased (11). Estrogen plays a key role in skeletal growth and bone homeostasis in men and women (12). Estrogen has bone and cellular receptors, and its effects are predominantly mediated by the action of these receptors (13). Effects of phytoestrogens are similar to estrogen since they also bind to estrogen receptors and activate some related signaling pathways (14). There are some reports about positive effects of phytoestrogens on bone health and mineral density (15). Fisetin is a phytoestrogen that is a component of the human diet, additionally, fisetin has anticancer, antioxidant and anti-inflammatory properties (16, 17). But, its effects on the differentiation of osteoblast have not been studied. Hence considering the effects of phytoestrogens on bone tissue and the differentiation of bone cells, as well as the specific structure of BC, in the present study 3D nanofibrous BC hydrogel scaffolds were prepared and loaded with fisetin and their ability to support and maintain the proliferation and osteogenic differentiation of rat BMSCs *in vitro* was investigated. 

## Materials and Methods

***Reagents***

For the present study, we purchased Dulbecco’s modiﬁed essential medium (DMEM) (Cat No: D5546), penicillin streptomycin (Cat No: P4333), 2,5-diphenyltetrazolium bromide yellow tetrazole (MTT) (Cat No: 298-93-1), fisetin (Cat No: F4043) from the Sigma–Aldrich company (USA). Na_2_HPO_4_ (Cat No: 1065860500), acid citric (Cat No: 1002415000), NaOH (Cat No: 1064621000) was obtained from Merck Co (Germany). In addition, peptone (Cat No: DF1810-08-0) and yeast extract (Cat No: BP1422-100), were obtained from Dif Co. High Pure RNA Isolation Kit (Cat No: 11 828 665 001) purchased from Roche Germany, cDNA Synthesis Kit (Cat No: K1622) and PCR Kit (Cat No: D204143) were purchased from Fermentas Inc, Germany. Primers were purchased from Bioneer (Korea), and the alkaline phosphatase activity measurement kit (Cat No: CBA-307), was purchased from Pars Azmoon Industry (Iran). All the solutions were prepared using double sterile distilled water. Other used chemicals were of analytical grades.

***BC preparation and morphological characterization***

The strain *Gloconacetobacter xlinus* (PTCC 1734) was commercially obtained from the Iranian Culture Center of Microorganisms. The bacteria were cultured in Hestrin-Schramm medium under static conditions at 30 °C for 7–10 days. The medium consisted of D-glucose (20 g/l), yeast extract (5 g/l), peptone (5 g/l), Na_2_HPO_4_ (2.7 g/l), and citric acid (1.5 g/l). To produce cellulose, the bacteria were diluted on Hestrin-Schramm media with a ratio of 1:10, and incubated in 24 well plates for 7 days. The thin layer of BC formed at the liquid–air interface. The BC scaffold was treated by boiling in 1 wt % sodium hydroxide for 2 hr at 90 °C to kill the bacteria. Subsequently, the membrane was thoroughly washed with running distilled water, after which it was soaked in 1 wt% aqueous sodium hydroxide solution for 24 hr at room temperature to eliminate the cell debris and components of the culture liquid. The pH was then reduced to pH=7 and stored in DI water at room temperature prior to use. Morphology of lyophilized BC scaffold was observed using a scanning electron microscope. For lyophilization, hydrated BC was placed in a −80 °C freezer for 24 hr and lyophilized in a freeze drier (FD10 Freeze Dryer, Mashhad, Iran). Dried BCs were fixed with 3% glutaraldehyde for 24 hr, rinsed 3 times with 25 mM PBS. Then samples were dehydrated through an alcohol series 30%, 50%, 70%, 90%, and pure acetone. The BC scaffolds were then dried in liquid CO_2_ and the BC surface was coated with gold (18). The surface morphology of the samples was observed using an SEM (LEO 1450VP FE-SEM; LEO, Germany) at 5 kV accelerating voltage.

***Cell culture study***

*BMSCs isolation, expansion and identification*

According to the previously published protocol described by Rahmanifar (19), BMSCs were isolated from femurs of rat (aged 6–8 weeks). The bone was cut and flushed by DMEM under sterile conditions. The cells were centrifuged and suspended in DMEM containing 10% FBS and incubated (37 °C in 5% humidified CO_2_ for 24 hr) until the medium was replaced to remove non-adherent cells. When the cells reached 90% confluency, they were trypsinized with 0.25% trypsin for five min at the temperature of 37 °C. After centrifugation, the cells were re-suspended in DMEM containing 10% FBS for further evaluation. To prove that the obtained cells were stem cells, flow cytometry was performed against the specific marker of the stem cells. The cells were centrifuged after separation from the bottom of the flask. One ml blocking buffer was added , cells were vortexed and incubated on ice for 30 min then centrifuged briefly, the supernatant was aspirated, and the cell pellet was resuspended with 125 μl FACS buffer containing diluted CD44, CD90 and CD45 primary antibodies conjugated with Fluorescein isothiocyanate (FITC) per manufacturer’s recommendations, then vortexed and incubated on ice for 60 min. The cells were washed with PBS, fixed with formalin and analyzed by a flow cytometer (BD, Dublin, UK) (20).

*BMSCs* *seeding and attachment on the BC scaffold*

The BC scaffold was transferred to the 24 well culture tissue plate and sterilized under UV light for 30 min. The cell suspension was added dropwise on the top of the scaffold at a density of 300,000 cells in 30 μl culture medium to allow for the initial cell attachment. Twenty min later, 170 μl of medium was added to the base of the plate to cover the scaffolds (21). After 4 hr of seeding cells, the culture medium was aspirated and the wells were washed with PBS three times to remove non-adherent cells. BMSCs attachment on the scaffolds was detected by SEM as described in the previous section.

*BC Biocompatibility using BMSCs*

BMSCs were used to assess the biocompatibility of BC. The cells were cultured in 96-well plates (6000 cells per well) and incubated with or without the BC scaffold and the proliferation of cells was quantified by MTT assay. For this assay, after 48 and 72 hr of incubation, 10 μl of MTT solution (5 mg/ml in PBS) was added to each well, which was left in the dark for 4 hr. The produced insoluble formazan was dissolved in 100 µl of DMSO and preserved at room temperature for 20 min. Finally, absorbance was read at 570 nm using a plate reader spectrophotometer (Epoch, Biotek, Winooski, VT, United States), and cell viability was calculated using the following equation (22).

Cell viability (%) = (A _treated_/A _control_) × 100

Where A _treated_ and A _control_ represent the absorbance of the treated and untreated cells, respectively. 

***Fisetin loaded BC scaffold, cell viability, and osteogenic differentiation***

*Cell viability*

BC scaffold was transferred into a 96 well culture plate and BMSCs (5× 10 ^4 ^per well ) were seeded on the BC scaffold and treated with 200, 400 and 800 µg/ml of fisetin for 48 hr. The cell viability was determined using MTT assay as described previously (22).

*Alkaline phosphatase (ALP) activity*

BMSCs (2 × 10^5 ^per well) were seeded on the BC scaffold in 12 well plates, as described previously. After 24 hr, samples were treated with 200, 400 and 800 µg/ml of fisetin for 10 days. To determine the level of ALP activity, total cell protein was extracted using 200 µl of the NP40 buffer. At the next stage, the lysate was centrifuged at 14,000×g at the temperature of 4 ºC for 15 min. The supernatant was collected and ALP activity was measured with the ALP assay kit. The kit uses p-nitrophenyl phosphate (pNPP) as a phosphatase substrate which turns yellow (λ_max_= 405 nm) when dephosphorylated by ALP (23). 

*Alizarin red staining (ARS)*

Based on the results of MTT and ALP activity assays, BMSCs (2 × 10^5 ^per well) were seeded on the BC scaffold in 12 well plates, as described previously; after 24 hr samples were treated with 800 µg/ml of fisetin for 21 days, the highest bone differentiation occurs over a period of 21 days. On the 21^st^ day, samples were washed with PBS and fixed with 4% paraformaldehyde at room temperature for 10 min, after washing once with distilled water. Next, sufficient ARS solution was added, in order to cover the cellular monolayer which was incubated at room temperature in a dark environment. The staining solution was removed after 15 min and each well was washed and observed using an inverted microscope (Bio med, Korea) (24).

*RNA extraction, cDNA synthesis, and real-time PCR*

BMSCs (30×10^6^) were treated with 800 µg/ml of fisetin for 21 days on the BC scaffold. After the treatment, RNA was extracted in accordance with the instruction of the manufacturer. Total RNA was extracted using Trizol reagent according to the manufacturer’s instructions. Additionally, the mRNA was reverse transcribed to cDNA using Advantage RT-for-PCR per manufacturer’s instructions. cDNA was amplified using a thermocycler (Perkin Elmer Applied Biosystems, Boston, MA) at 94 ^°^C for 40 sec, 56 ^°^C for 50 sec, and 72 ^°^C for 60 sec for 35 cycles, after initial denaturation at 94 ^°^C for 5 min. Real-time PCR was carried out using a Bio Rad Detection System (Bio Rad, US), specific primers (Table 1) and SYBR green dye. Reactions were run in triplicate in three independent experiments. The geometric mean of housekeeping gene GAPDH was used as an internal control to normalize the variability in expression levels (22).

***Statistical analysis***

Data analysis was performed in SPSS version 16.0 and the statistical significance was determined using one- or two-way analysis of variance and Tukey’s multiple comparison test. In all analyses, a 95% confidence level (*P*<0.05) was considered statistically significant. All the data were presented as mean ± standard deviation (SD).

## Results

***BC morphological characterization***

**Figure 1:**
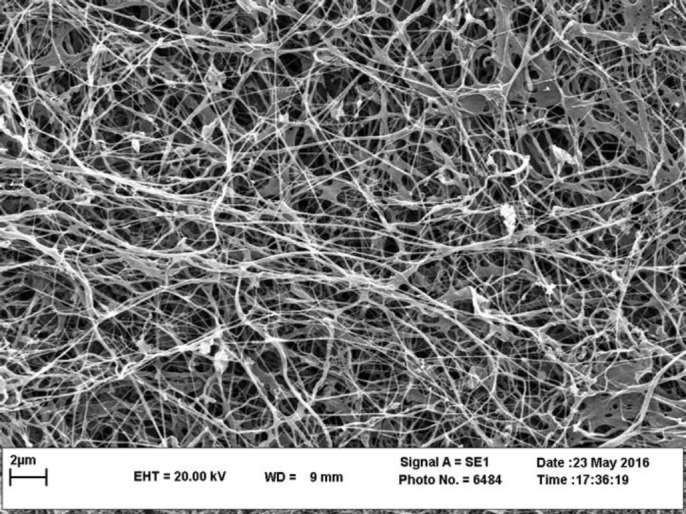
Morphological observation of BC structure by SEM. The BC was freeze-dried then sputtered with gold and photographed. SEM image of BC, aligned and porous structure of BC nanofibers are shown. Fiber of bacterial cellulose diameter is found to be between 20–60 nm

The fiber structure of BC was visualized using SEM. Accordingly, BC was observed to have refined three-dimensional network nanostructures, with the fiber diameters of at nano-level (Figure 1).

**Figure 2:**
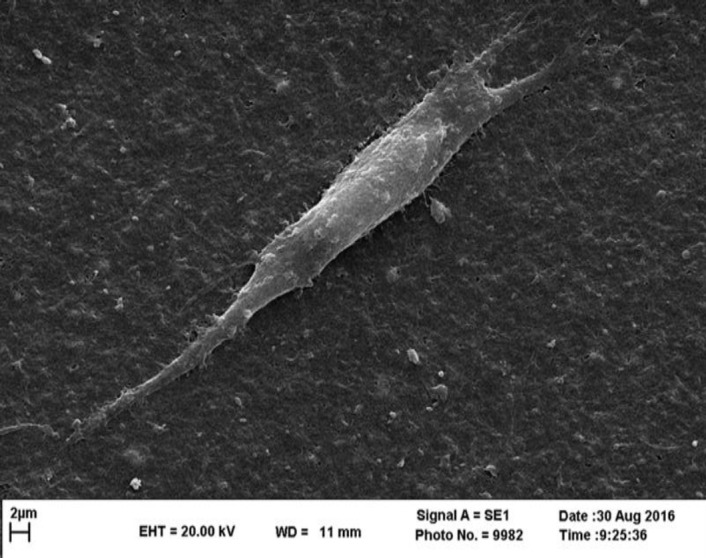
Cell adhesion on BC scaffold. BMSCs were seeded on the BC scaffold, the adhesion and morphology of BMSCs on this scaffold were monitored using SEM. Images showed BMSCs attachment to the BC scaffold. Results indicated good cell adhesion and proliferation

Image of SEM showed BMSCs adhesion to the BC scaffold. They showed BC is an appropriate environment for BMSCs like ECM (Figure 2).

***Stem cell identification***

**Figure 3:**
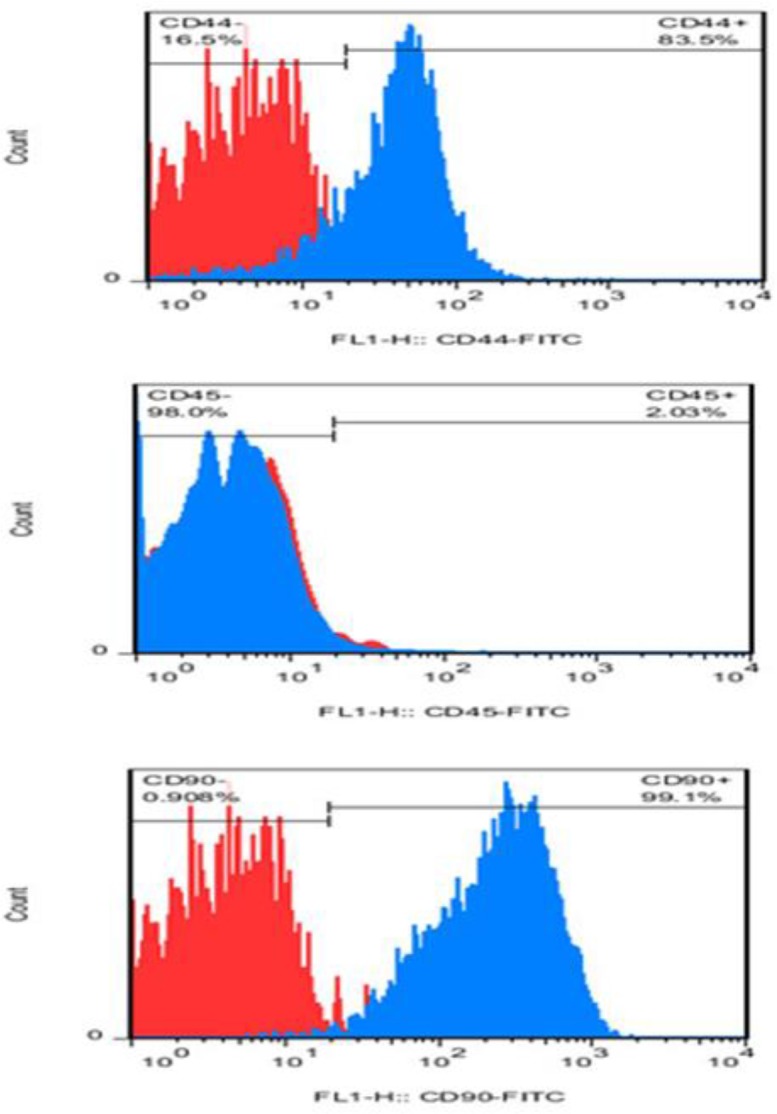
Flow cytometry results for mesenchymal stem cells identification: BMSC surface marker CD44 and CD90 was expressed to levels greater than 83% and 99%, while expression of hematopoietic marker (CD45) was less than 3%

Flow cytometry results confirmed the presence of mesenchymal stem cells. Furthermore, results of flow cytometry analysis regarding the isolated BMSCs for the mesenchymal stem cell marker (CD44), hematopoietic marker (CD45) and endothelial marker (CD31), were positive for CD44, while they were negative for CD31 and CD45 (Figure 3).

**Figure 4:**
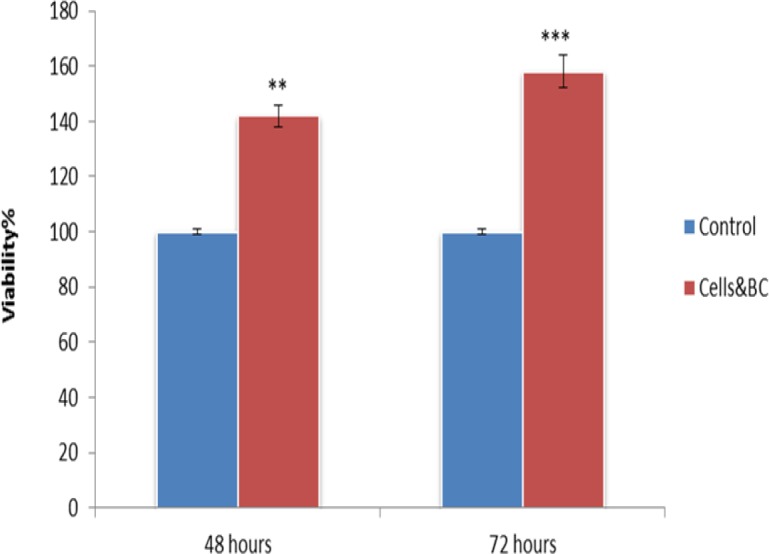
BC scaffold is BMSCs biocompatible. Cells cultured in DMEM with the BC scaffold (as treated groups) and without BC (as control groups). Results showed BC doesn’t have any toxic effects on BMSCs (**P<0.01, ***P<0.001)

**Figure 5:**
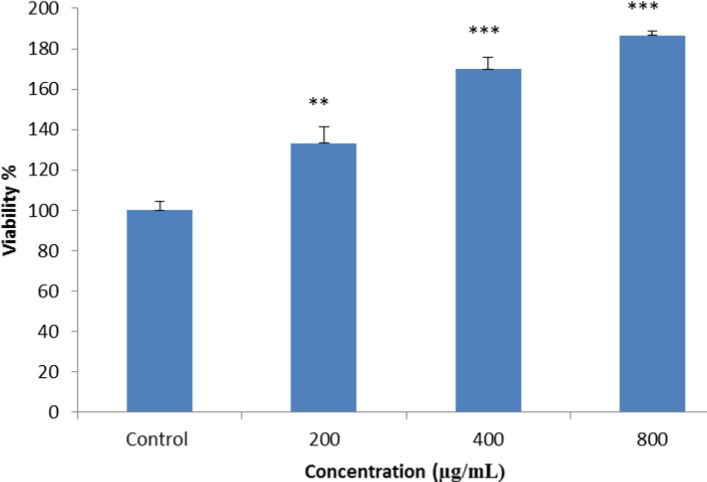
MTT assay was performed to evaluate potential cytotoxic effects of fisetin on BMSCs. The cells were seeded on a BC scaffold and treated with 200, 400, and 800 µg/ ml of fisetin for 48 hr, and cell viability was measured, using MTT assay. The results indicated that the viability of cells increased dose-dependently (**P<0.01, *** *P*<0.001)

**Figure 6.**
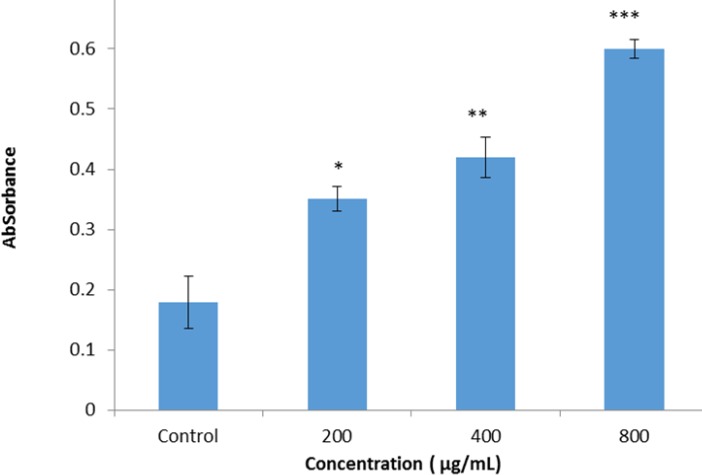
The ALP activity was detected with alkaline phosphatase substrate p-NPP. The cells were seeded on a BC scaffold and treated with 200, 400, and 800 µg/ ml of fisetin for 48 hr, and ALP activity on BMSCs that were seeded on BC scaffolds loaded with fisetin in comparison with BMSCs seeded on BC without fisetin was exanimated. The result showed ALP activity increased dose-dependently in BMSCs seeded on BC in the presence of fisetin compared with the BMSCs on tissue culture without fisetin (*P<0.05, **P<0.01, ***P<0.001)

**Figure 7:**
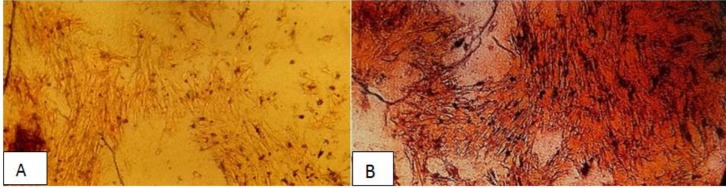
Alizarin red staining of calcium accumulation in BMSCs cultured on the scaffold in the presence of fisetin. (A) control group, the cells were seeded on BC without fisetin, (B) cells seeded on BC loaded with 800 µg/ml of fisetin. The result showed calcium deposition in the extracellular matrix is higher in groups that were seeded on BC load with the fisetin group. Magnification 10×10

**Figure 8:**
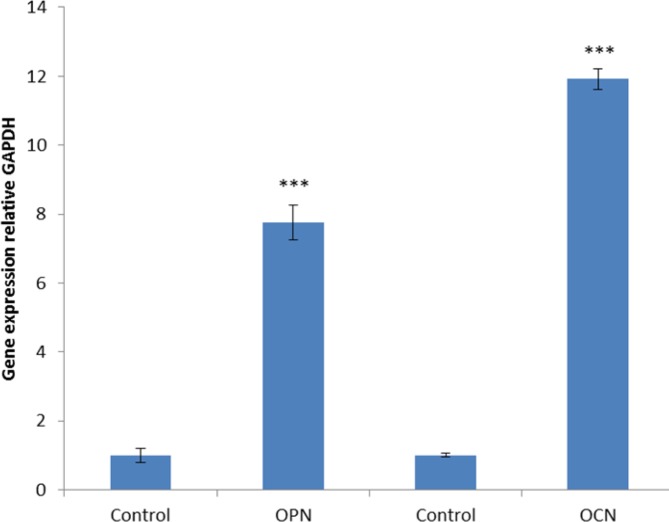
Gene expression analysis by real-time PCR. BMSCs were seeded on a BC scaffold loaded with 800 µg/ml of fisetin for 21 days in compression with BMSCs seeded on BC without fisetin as the control group. The expression of osteoblastic genes OPN and OCN significantly increased in BC scaffold loaded with fisetin in comparison with controls (***P <0001)

**Table 1:** Primers used for osteogenic differentiation

Gene	Forward	Reverse
GAPDH	5ʹTGAAGGTCGGTGTGAACGGATTTGGC3ʹ	5ʹCATGTAGGCCATGAGTCCACCAC3ʹ
OCN	5ʹGTGCAGAGTCCAGCAAAGGT3ʹ	5ʹ CGATAGGCCTCCTGAAAGC3ʹ
OPN	5ʹACAGCCAGGACTCCATTGAC3ʹ	5ʹACACTATCACCTCGGCCATC3ʹ

***BC biocompatibility***

BMSCs were cultured in DMEM medium with or without the BC scaffold and the viability of the cells was quantified using the MTT assay. The rate of cell viability in the BC group was significantly higher compared to the control group at 48 and 72 hr. Moreover, BC scaffold was observed to have biocompatibility (Figure 4).

***Cell viability and osteogenic differentiation***

*MTT assay*

According to the results of cytotoxicity, cell viability in the treated cells increased in a dose-dependent manner, while higher viability was observed in the cells treated with higher doses of fisetin (800 µg/ml) (Figure 5).

*ALP activity*

ALP enzyme activity after 10 days was matured within samples that were seeded on the BC scaffold in presence of fisetin. The results of ALP activity assay indicated that in cells seeded on the BC loaded with fisetin the absorbance at 405 nm was increased dose-dependently, compared to the cells seeded on BC without fisetin. The higher ALP activity was observed in 800 µg/ml of fisetin (Figure 6). Increase in the absorbance at 405 nm indicted the increased ALP activity.

*ARS*

In the current research, ARS was performed after 21 days on the cells on the scaffold, which were treated with 800 µg/ml of fisetin. According to the results, fisetin could induce the differentiation of BMSCs into osteoblasts on the BC scaffold. On the other hand, the BMSCs that received no fisetin treatment were considered as controls and the results indicated that the BC scaffold in the control group could contribute to BMSC mineralization (Figure 7).

*Real-time PCR*

 According to real-time PCR findings of this study, significant up-regulation of OPN and OCN expression occurred in the group treated with 800 µg/ml of fisetin. Furthermore, OPN and OCN genes expression were associated with osteoblast differentiation (Figure 8).

## Discussion

The aim of the study was investigating osteogenesis of BC loaded with fisetin as a scaffold for BMSCs. Obtained results indicated BC loaded fisetin differentiated BMSCs more efficiently than BC alone. Bone tissue engineering has mainly focused on finding functional treatments for bone defects(25). Functional treatments are based on the three main bone components of bone progenitor cells, bone growth factors and scaffolds for cell adhesion and maintaining bone performance (26). Normally a scaffold is used as a three-dimensional extracellular matrix in tissue engineering for the constant preservation of the cells (27). Some of the prominent features of scaffolds include mechanical strength, biocompatibility, biodegradability and proper porosity (28, 29). BC is a natural nano-fiber scaffold, which is formed by combining with nanocellulose and has high-biocompatibility (30). High biocompatibility of BC contributes to the reduction of the tissue’s inflammatory response (31). BC contains large amounts of fiber in its structure, which stimulates cell growth and attachment (5). Furthermore, the linear structure of BC facilitates the transfer of nutrients and oxygen (28, 32). BC has been investigated in previous studies as an appropriate scaffold in the tissue engineering of cartilage and bone (8, 29). Hence in the present study, we used BC as a scaffold for BMSCs differentiation. Also in tissue engineering, cell selection is of paramount importance. BMSCs are considered as one of the most appropriate cells in this regard, which are obtained quickly and easily and could differentiate into mesenchymal cell lineages such as osteogenic, adipogenic, myogenic, and chondrogenic (33). In this study, BMSCs were used as a cell source for investigating BC scaffolds loaded with fisetin. The cells were seeded on the scaffold, the morphology and cell adhesive properties of obtained BC were observed by SEM. Resulting image from SEM showed that the BC scaffold has a fibrous structure and BMSCs attach to the BC scaffold. Biocompatibility of BC was determined with the MTT assay after 48 and 72 hr, the results indicated BMSCs in presence of BC scaffold have the highest viability rate in comparison with the control group. Therefore, BC is biocompatible and does not exert toxic effects on BMSCs. Stimuli factors are a major aspect of tissue engineering. Cells could be stimulated into osteoblast differentiation with some osteogenic substrates. Fisetin is one of the phytoestrogens isolated from strawberries, apples, persimmons, grapes, mangos, onions, and cucumbers (34). Numerous studies have confirmed that phytoestrogens are able to induce osteogenesis *in vivo* and *in vitro* (35, 36). So in the current study, fisetin was used as an osteoblast inducer. To determine the appropriate dose of fisetin for induced differentiation we used the MTT assay.

According to the results of the present study 200–800 µg/ml of fisetin increased cell viability. Different reports shows the effect of plant extracts on cell viability. Mollazadeh *et al.* (2017) indicated that *Sophora pachycarpa* root extract had no toxic effect on cell viability and increases osteogenic differentiation in adiopose - derived human MSCs (37). One of the most efficient methods for the investigation of osteogenesis is ALP enzyme activity assay. Expression of this enzyme occurs in hard tissues, especially in mineralized tissue cells, the activity of the enzyme increases within days 10–14 of osteogenesis (38). In this study, ALP measurement was used to determinate BMSCs differentiation. Our findings suggested that 800 µg/ml of fisetin has the most significant effect on alkaline phosphate activity on the 10^th^ day of osteogenesis. 

Calcium is the essential bone mineral, and one of the techniques for determining osteogenesis involves the detection of calcium, ARS is used to verify bone mineralization, which has been used for decades to evaluate calcium-rich deposits by cultured cells (39). 

According to the results from current research 800 µg/ml of fisetin led to the deposition of calcium in the bone formation on the BC scaffold. Bone formation is a complex process associated with the expression of several specific genes. In the present study, we investigated the expression of osteocalcin and osteopontin which are the specific genes in the bone tissues (40). Osteocalcin is a late-stage osteoblast-specific protein, produced by mature osteoblasts only, osteopontin is the protein responsible for the expression of various tissue types, including preosteoblasts, osteoblasts, and osteocytes (41). Findings of the current study regarding gene expression indicated that 800 µg/ml of fisetin led to the expression of osteocalcin and osteopontin (*P*<0.001) (Figure 8).

Shi *et al.* (2012), prepared the BC scaffold from a *Gloconacetobacter xlinus* and investigated the osteogenic potential of the BC scaffold coated with bone morphogenetic protein-2 (BMP-2). The data confirmed that BC had a good biocompatibility and encouraged differentiation of mouse fibroblast-like C2C12 cells into osteoblasts in the presence of BMP-2 *in vitro*, as shown by ALP activity assays. In *in vivo* subcutaneous implantation research, BC scaffolds carrying BMP-2 displayed more bone formation and higher calcium concentration than the BC scaffolds alone. Their studies indicate that BC is a good localized delivery system for BMPs and possibly a potential candidate in bone tissue engineering (7). Moreover, Zaborowska and co-workers stated that microporous BC is able to mimic the bone tissue and is a potential scaffold for bone regeneration. They seeded BC with NC3T3-E1 osteoprogenitor cells and investigated osteogenesis by ARS and histological test (8). Hydroxyapatite was reported to cause the feasibility of BC scaffold to support osteoblast growth and bone formation. Furthermore, the researchers investigated the osteogenesis of SaOS-2, an osteoblast-like cell line by ALP activity and ARS (42). In 2013, Favi *et al.* reported that the multipotent MSCs on natural biopolymers could hold a great promise for the treatment of connective tissue disorders, such as osteoarthritis. Moreover, BC a biocompatible natural polymer was assessed, as a scaffold for equine-derived bone marrow mesenchymal stem cells (Eq-MSCs) for the applications of bone tissue engineering *in vitro*. It is noteworthy that ALP activity and ARS assays were used for the detection of osteogenesis (43). Qin *et al.* investigated the effects of icaritin and composite scaffolds on BMSCs. According to the *in vitro* tests, icaritin enhanced the expression of osteogenesis-related genes, such as COL1a, osteocalcin, RUNX2, and BMP-2, on a poly (lactic-co-glycolic acid)/tricalcium phosphate (P/T) scaffold (44). Another research in this regard evaluated the bone formation potential of a polycaprolactone scaffold loaded with resveratrol phytoestrogen. Results of ALP activity assay and Von Kossa staining showed that resveratrol improved bone formation in BMSCs compared to the control group receiving no resveratrol (45). In a similar study, effects of resveratrol treatment on the osteogenic potential of adipose-derived stem cells in a PCL/collagen 3-D culture environment were investigated (46). In this study it was shown that fisetin as a stimuli factor can enhance the osteogenic differentiation of BMSCs on BC scaffolds, these results are consistent with another study. 

## Conclusion

Findings of the present study confirmed the ability of the BC scaffolds loaded with fisetin to support BMSC attachment, proliferation, and bone-specific matrix biosynthesis.
